# A novel “complement–metabolism–inflammasome axis” as a key regulator of immune cell effector function

**DOI:** 10.1002/eji.201546131

**Published:** 2016-06-08

**Authors:** Giuseppina Arbore, Claudia Kemper

**Affiliations:** ^1^ MRC Centre for TransplantationDivision of Transplant Immunology and Mucosal BiologyKing's College LondonLondonUK; ^2^ Laboratory of Molecular Immunology and the Immunology CenterNational Heart, Lung, and Blood Institute (NHLBI)National Institutes of Health (NIH)BethesdaMDUSA

**Keywords:** Complement, Metabolism, NLRP3 inflammasome

## Abstract

The inflammasomes are intracellular multiprotein complexes that induce and regulate the generation of the key pro‐inflammatory cytokines IL‐1β and IL‐18 in response to infectious microbes and cellular stress. The activation of inflammasomes involves several upstream signals including classic pattern or danger recognition systems such as the TLRs. Recently, however, the activation of complement receptors, such as the anaphylatoxin C3a and C5a receptors and the complement regulator CD46, in conjunction with the sensing of cell metabolic changes, for instance increased amino acid influx and glycolysis (via mTORC1), have emerged as additional critical activators of the inflammasome. This review summarizes recent advances in our knowledge about complement‐mediated inflammasome activation, with a specific focus on a novel “complement – metabolism – NLRP3 inflammasome axis.”

## Introduction

The immune system constantly orchestrates a range of effector pathways to handle the detection and elimination of not only exogenous microbes but also endogenous dangerous entities, such as infected, apoptotic or malignant cells. Microbe‐derived molecules, known as pathogen‐associated molecular patterns (PAMPs) and noxious self‐derived molecules, named danger‐associated molecular patterns (DAMPs), are recognized by different pattern recognition receptors (PRRs) expressed on or secreted by immune cells. Among these ‘systems’ comprising classic PRRs, are the toll‐like receptors (TLRs), the Nod‐like receptors (NLRs) and the retinoic acid inducible gene 1 (RIG‐I) receptors, and several proteins of the complement system (reviewed in 1, 2). The engagement of these PRRs leads to cell activation and induction of appropriate protective effector functions specific for the cells that receive these signals. One of the most critical effector functions of the PRRs is to induce assembly and activation of “inflammasomes.” Inflammasomes are multiprotein complexes, which are required for the induction and maturation of the key host proinflammatory cytokines IL‐18 and IL‐1β. In particular, the engagement of TLRs, NLRs, and RIG‐Is have been demonstrated to activate the inflammasomes via a series of different downstream pathways (reviewed in 1, 3).

Recent work, however, demonstrates that activation of the complement system and subsequent engagement of complement receptors and regulators on immune cells, either independently or in conjunction with incoming signals from TLRs or RIG‐Is, are also vital signals for the activation and optimal function of inflammasomes 4, 5. Given that the complement system is evolutionarily among the oldest PRR systems 2, and that extensive cross‐talk between complement and particularly the TLRs exists 2, 6, the involvement of complement in inflammasome activation is not unexpected. What comes as a surprise, however, is that the complement system emerges as central regulator of basic metabolic processes of the cell and that a novel “complement—metabolism–inflammasome axis” may be contributing to optimal effector function of immune cells during inflammation.

In addition to specific danger sensors such as the PRRs, both innate and adaptive immune responses are also modulated by a series of metabolic checkpoint systems. For example, quiescent cell subsets, such as nonactivated and circulating myeloid and lymphoid cells, rely on mitochondrial oxidative phosphorylation (OXPHOS) via the tricarboxylic acid cycle and beta‐oxidation of fatty acids, as their main energetic fuel 7, 8, 9. Conversely, activation of such cells and induction of effector function requires, in most cases, increased glycolysis to supply the elevated energy demand for proliferation, induction of cytokine secretion, phagocytosis etc. 7, 8, 9. Importantly, these metabolic changes, including increased glycolysis, mitochondrial stress, and membrane electrolyte fluxes, are sensed within the cell and have been shown to subsequently activate and/or modulate particularly NLRP3 inflammasome functions 3, 10, 11, 12. Conversely, it has been demonstrated that several complement receptor/regulators drive the regulation of nutrient channel expression and influx of amino acids (AAs) and glucose, as well as direct regulation of glycolysis and OXPHOS levels (at minimum in CD4^+^ T cells) 13, and complement is now increasingly being connected with the metabolic reprogramming required for immune cell effector function. In this short review, we will discuss insights into the emerging novel functional relationships between the complement system, key cell metabolic pathways, and inflammasome activation and function.

### The inflammasome

Upon specific induction signals, including exogenous antigens derived from invading microbes or endogenous ligands generated during cell activation and/or stress 1, 3, several members of the NLR protein family, the Pyrin domains‐containing protein (NLRP) family and the Pyrin and HIN domain containing (PYHIN) family form multiprotein complexes known as “inflammasomes,” whose structure and functions are conserved in vertebrate species 14. Members of the NLR protein family include NAIP (neuronal apoptosis inhibitor protein), C2TA (class 2 transcription activator, of the MHC), HET‐E (heterokaryon incompatibility) and TP1 (telomerase‐associated protein 1), leucine‐rich repeats, and Pyrin domains‐containing protein 1 (NLRP1), NLRP3, NLRP6 as well as NLR family CARD (caspase activation and recruitment) domain containing 4 (NLRC4), and members of the PYHIN family include absence in melanoma 2 (AIM2) and IFN‐γ inducible protein 16 (IFI16) 3, 15, 16. These different inflammasomes all respond to different danger signals (reviewed in 3, 15). Currently, the best‐characterized inflammasome both in humans and mice is the NLRP3 inflammasome complex, which is composed of NLRP3 (known also as cryopyrin, NALP3, CIAS1, or PYPAF1), the adaptor apoptosis speck protein (ASC), and procaspase‐1 17. NLRP3, as shown for other NLRs, is present in the cytoplasm in an inactive form associated with the heat shock protein 90 (HSP90) and the ubiquitin‐ligase suppressor of the G2 allele of skp1 (SGT1) 18. Sensing of specific molecules, such as bacterial peptidoglycans or the bacterial cell wall component LPS by TLR4 or monosodium urate particles, induce inflammasome activation: specifically, HSP90 and SGT1 release NLRP3, which then binds the Pyrin domain of ASC, leading to procaspase‐1 binding to the ASC CARD domain, via CARD–CARD homotypic interactions 3, 15, 16, 18. This binding cascade triggers the formation of oligomers (speck complexes) of many molecules of NLRP3, ASC, and procaspase‐1, with this active complex then releasing the functional “end product,” the active p20 and p10 caspase‐1 fragments 1, 15. Noncanonical NLRP3 inflammasome activation (which is independent from surface TLR4 engagement by LPS, but triggered instead by cytosolic sensing of LPS derived from bacteria that have escaped the phagolysosome) can also occur either via caspases‐4/5 19, 20, caspase‐8 21, and caspase‐11‐mediated assembly of the NLRP3 inflammasome complex 22. However, there is currently no known functional connection between complement and caspase activation, and the focus in this review will thus be on the functional connection between complement and canonical inflammasome activation. The main function of NLRP3 inflammasome‐driven caspase‐1 activation is the subsequent conversion of inactive proIL‐1β and proIL‐18 into their active and secreted forms 3. IL‐1β and IL‐18 are proinflammatory cytokines and required for protective innate and adaptive immune responses. IL‐1β and IL‐18 are mostly produced by monocytes, macrophages, APCs, and neutrophils, and enhance the effector function of these cells during pathogen clearance (reviewed in 23). Furthermore, it has been shown that IL‐1β supports T‐cell priming and Th1 and Th17 responses in humans 24, 25 and memory T‐cell generation in mice 26, while IL‐18 enhances IFN‐γ production by NK cell T lymphocytes, as demonstrated in mouse studies 27.

NLRP3 inflammasome function has been described both in human and in mice in myeloid innate immune cells, with monocytes as the main source of IL‐1β 25, 28, and in several nonimmune cell types (such as microglia, endothelial, and retinal pigment epithelial cells) in both species 29, 30, 31.

Although the generation of IL‐1β is critical to combat invading pathogens, uncontrolled inflammasome activation has also been demonstrated to contribute to the development of pathogenic (auto) inflammatory diseases, including type 1 diabetes and rheumatoid arthritis (RA) 32, 33. For example, gain‐of‐function mutations in NLRP3 are associated with a group of heritable monogenic syndromes known as cryopyrin‐associated periodic syndromes (CAPS), characterized by excessive production of IL‐1β from APCs with recurrent fevers, skin rashes, joint and ocular inflammation, and amyloidosis in patients 34. Therapeutic suppression of the unwanted inflammatory responses in CAPS patients can be achieved by IL‐1R blockade with the IL‐1 receptor antagonist anakinra, or canakinumab, a monoclonal antibody targeting IL‐1R1 34, 35. Thus, NLRP3‐driven IL‐1β secretion is tightly regulated and requires, at minimum, two “danger” signals: Signal 1 is a priming signal and is initiated by the engagement of other PRRs, including TLR4 or by cytokine receptors, such as the tumor necrosis factor receptor, which then promote nuclear translocation of nuclear factor κB (NF‐kB), leading to gene transcription and translation of *NLRP3* and *IL1B* 36. Several exogenous or endogenous molecules can deliver Signal 2, which induces NLRP3 assembly and formation of ASC speck aggregates. Exogenous signals inducing NLRP3 assembly include microbial, fungal, viral, and parasitic products such as toxins and glycans and environmental polluters such as silica and asbestos 3, 37, 38, 39 and disease‐associated accumulation of altered protein complexes, such as cholesterol crystals in atherosclerosis, amyloid β in Alzheimer disease and monosodium urate in gout 3, 5, 29. Endogenous sources of Signal 2 include products of events occurring during cell activation, including reactive oxygen species (ROS), cathepsins B and L released during lysosomal damage, increased cytoplasmic Ca^2+^ as a product of ion fluxes (K^+^ efflux), altered adenosine triphosphate (ATP) production or ATP influx via the ATP receptor P2X purinoceptor 7 (P2X7), and changes in, glucose and lipid metabolism [3, 10, 11, 12, 40]. The common denominator among the endogenous signals driving NLRP3 inflammasosme activation is that they are all derived from metabolic changes accompanying cell (hyper) activation. Thus, NLRP3 appears to be a major sensor for cell metabolic activity and stress, and this is underscored by the recent observations that dysregulation of the NLRP3 inflammasome contributes also to metabolic diseases and pathologies, such as gout, type 2 diabetes (T2D), nonalcoholic fatty liver disease, obesity, and cancer (reviewed in 34).

There are several excellent reviews published on these new pathways driving NLRP3 inflammasome activation and function for further in‐depth reading 41, 42.

### The complement system

The complement system was originally defined over a century ago by Paul Ehrlich as a “system” of serum‐circulating proteins able to “complement” the antibody‐mediated and cell‐mediated immune responses 43. Complement is a critical part of innate immunity and a major initiator of the inflammatory reaction. It is comprised of over 50 blood‐circulating, mostly liver‐derived, and membrane‐bound proteins and regulators with the effector molecules existing largely in a precursor state that is activated rapidly in a cascade‐like fashion following activation of the system 44. Three pathways lead to complement activation in blood: the classical, the lectin, and the alternative pathway (reviewed in 44) and all pathways are triggered when complement sentinel proteins, such as the mannose‐binding lectin (MBL) and C1q 45, 46 sense the presence of microbes. Importantly, and similar to TLRs and the inflammasomes, complement‐derived sensors recognize both PAMPs but also DAMPS. For example, MBL binds to specific carbohydrates only present on pathogens such as mannose 45, while ficolins and the C1 complex (C1q) detect danger molecules produced by stressed and dying cells, such as surface blebs on apoptotic human keratinocytes 46. Although triggered by different signals, all activated pathways—the classical, the lectin, and the alternative—cumulate into the generation of a “so‐called” C3 convertase, a protein complex that cleaves the key complement effector molecule C3 into the bioactive opsonin C3b and the anaphylatoxin C3a. The subsequent generation of C5 convertases activates C5 into C5b (which seeds the generation of the pore‐forming membrane attack complex (MAC)) and the anaphylatoxin C5a 44. Functionally, the activation of complement leads to the opsonization of targets (via C3b), the migration and activation of innate immune cells (via the receptors for the anaphylatoxins C3a and C5a) and direct lysis (via the MAC) and/or phagocytic uptake of the target pathogen by scavenger cells (via engagement of C3 activation fragment receptors) 47.

Aside from the mobilization of innate immune cells and induction of the general inflammatory reaction, complement also directly modulates adaptive immunity 44. Engagement of complement receptor 2 (CR2, CD21) through C3d‐coated antigen during BCR activation in the lymph nodes has been shown to reduce the threshold for BCR signaling, and is needed for optimal antibody production in mice 48. Also, macrophages capture immune complexes that are recognized by follicular B cells in the subcapsular sinus via CR2 48, 49, and, in addition, immune complexes coated with C3 activation fragments are recognized by follicular dendritic cells, which retain the antigen, thus enhancing B‐cell memory and effector differentiation in the germinal centers 50. Complement activation products also impact on T‐cell activation and regulation, either indirectly by influencing the maturation and function of APCs, or directly by engaging complement receptors/regulators expressed on T cells 51. For example, concurrent signaling by the C3b/C4b‐binding complement regulator CD46 (membrane cofactor protein, MCP) and the C3aR expressed on CD4^+^ T cells has been proved to be a prerequisite for IFN‐γ production and human Th1‐cell induction 52, 53, 54, and dysregulation of CD46‐mediated costimulatory signals on T cells has been identified in chronic disease settings, including multiple sclerosis 55 and RA 52. Moreover, in mice (which do not express CD46 on somatic tissue 44), the anaphylatoxin receptors are shown to be critical for the induction and regulation of Th1, Th2, Th17, and natural Treg‐cell responses 56, 57.

Specifically, the recent work on the function of complement receptors and regulators on human CD4^+^ T cells has led to two important new discoveries: First, immune cells produce complement C3 and C5 activation fragments that in turn engage cell‐expressed complement receptors in an autocrine fashion (thus, providing signals independent of liver‐derived serum‐circulating complement) 51, 54, 57 and, second, complement activation is not confined to the extracellular space but occurs intracellularly [52, 54, 58]. Importantly, engagement of intracellular complement receptors, such as C3aR, induces signaling pathways that are distinct from those induced by the same complement receptors expressed on the cells surface 44, 54. Specifically, C3 activation occurs intracellularly via cathepsin L‐mediated cleavage of C3 in human CD4^+^ T cells, and the C3a generated via this “pathway” has been shown to stimulate intracellular C3aR signaling on lysosomes; this intracellular C3aR signaling in turn drives the homeostatic survival of resting T cells via tonic mammalian target of rapamycin (mTOR) stimulation 54. During TCR activation in human T lymphocytes, C3a and C3b generated intracellularly rapidly translocate to the cell surface where they engage cell surface C3aR and CD46, respectively, both of which are events driving productive IFN‐γ secretion 13, 54. In addition, experimental observations demonstrate that intracellular C3 activation by cathepsin L is dysregulated in T cells from patients with RA, and contributes to Th1 hyperactivity, but can be normalized via a cell‐permeable cathepsin L inhibitor 54.

A more detailed description of extra‐ and intracellular complement activation, regulation, and function in mice and humans are thoroughly reviewed in 44.

### Complement in the activation and regulation of the NLRP3 inflammasome

**Figure 1:**
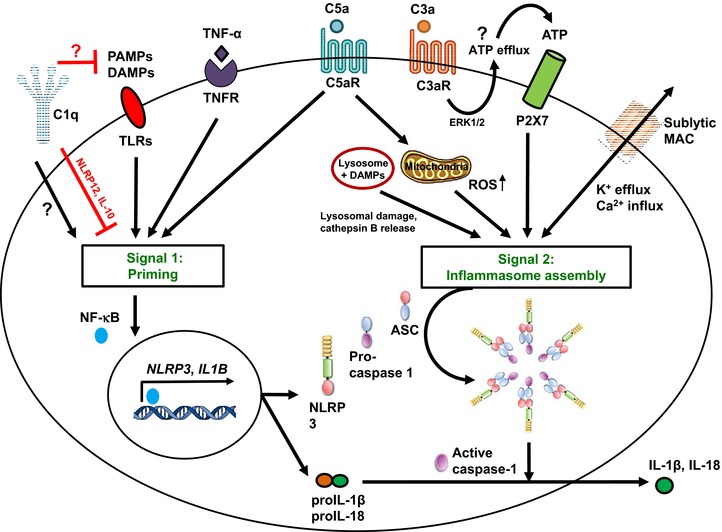
Mechanisms of complement‐mediated NLRP3‐inflammasome regulation. NLRP3 inflammasome activation requires two distinct signals. The “priming” Signal 1 is triggered by PAMP/DAMP recognition by PPRs (e.g. TLRs) and certain cytokines (TNF‐α) and drives NF‐κB nuclear translocation and *NLRP3* and *IL1B* gene transcription. Signal 2 induces the assembly of NLRP3, ASC, and caspase‐1 supracomplexes to form an active NLRP3 inflammasome, where active caspase‐1 processes proIL‐1β/proIL‐18 into mature IL‐1β/IL‐18. The complement components C1q and C5aR1 (together with tumor necrosis factor receptor and/or TLR signaling) potentiate Signal 1. C5aR1 act as a priming signal to sustain inflammasome activation during the uptake of DAMPs, with a mechanism involving increased lysosomal damage and cathepsin B release. C5aR1 activation directly delivers Signal 2 for NLRP3 inflammasome activation, via induction of mitochondrial damage and intracellular accumulation of ROS. The C3aR regulates ATP efflux (via a not yet identified channel, denoted by a question mark) and subsequent autocrine P2X7 engagement, and sublytic MAC formation increases intracellular Ca^2+^ levels and mitochondrial membrane potential. Of note, C1q can increase canonical NLRP3 inflammasome activation in epithelial cells through a not yet defined mechanism (denoted by a question mark) but can also function as a negative regulator of NLRP3 inflammasome activation by sequestering DAMPs (such as cholesterol crystals) and inhibiting PPR signaling.

Given that the complement system and the NLRP3 inflammasome share a “mutual interest” in protecting the host against danger, a functional cross‐talk between these two systems feels natural (Fig. 1). Indeed, studies in the 1980s by Haeffner‐Cavallion et al. showed that the anaphylatoxin C3a induces IL‐1β production in human monocytes 59, indicating a functional connection between these two systems at a time before the inflammasome was actually discovered. A more recent study defined the C3aR‐driven signals in human monocytes and demonstrated that locally produced C3a increases ATP efflux from the monocyte cytosol (via ERK1/ERK2 phosphorylation and expression regulation of a yet‐unidentified channel) in the presence of TLR4 activation by LPS 4. This C3a‐driven ATP efflux leads to subsequent increased activation of the ATP receptor P2X7, a potent Signal 2 for NLRP3 inflammasome activation 37, and substantially increased IL‐1β secretion 4 (Fig. 1). Furthermore, the anaphylatoxin C5a has been confirmed as an important driver of Signal 1 for NLRP3 inflammasome activation in human monocytes: Samstad et al. 5 showed that cholesterol crystals activate both the classical (via C1q) and alternative complement pathways and that C5a generated during this process, together with TNF‐α, functions as priming Signal 1 for NLRP3 activation by increasing *IL1B* gene transcription (Fig. 1). Work by the same group has determined that high‐density lipoprotein reduces complement activation on cholesterol crystals and leads to a reduction in NLRP3 inflammasome activity in monocytes and granulocytes during atherosclerosis 60. C5aR‐mediated signals may regulate *IL1B* transcription via NF‐κB activation because locally increased C5a levels have been shown to induce NF‐κB activation in retinal pigment epithelial cells and trigger IL‐18 secretion from these cells 31. In mouse retinal epithelial cells, C1q also increases NLRP3 inflammasome‐dependent IL‐18 production (which protects unexpectedly against the progression of age‐related macular degeneration) in mice, however, the exact mechanism but which this occurs remains to be identified 61. There is also indication that CD46 partakes in NLRP3 inflammasome priming, as CD46 engagement during TCR stimulation on human CD4^+^ T cells potentiates NF‐kB activation 13 and increases transcription of *IL1B* [58]. C5a activation can also increase inflammasome activation in models of gout disease: C5aR1 activation on monocytes, primes, and potentiates NLRP3 inflammasome activation induced by uric acid crystals, with the latter being dependent on lysosomal damage and cathepsin B activity 62 (Fig. 1); this role for C5a as has been further corroborated in neutrophils in a mouse peritonitis model and in human gout exudates from the articular joints 63.

One of the other major routes by which C5a (and possibly C3a) may regulate NLRP3 inflammasome activity is likely via increased generation of ROS [5, 58]. ROS not only constitutes a general danger Signal 2 for inflammasome activation 15, 64, but has traditionally also been linked tightly with anaphylatoxin receptor activation in APCs, neutrophils, and granulocytes 5, 65, 66. Furthermore, C5aR1 activation involves PI3 kinase signaling 56, which has been placed upstream of mitochondrial ROS production and NLRP3 inflammasome activation 67 (Fig. 1).

In line with the fact that pore forming toxins are strong inflammasome inducers 37, sublytic deposition of the MAC has been demonstrated to activate the NLRP3 inflammasome in murine dendritic cells and elicits IL‐1β and IL‐18 secretion in a caspase‐1‐dependent fashion 68. Similarly, in primary human lung epithelial cells, sublytic MAC induces the NLRP3 inflammasome via increased intracellular Ca^2+^ fluxes from the endoplasmic reticulum, Ca^2+^ accumulation in the mitochondrial matrix and loss of mitochondrial transmembrane potential (Signal 2) that triggers the NLRP3 inflammasome 69, 70 (Fig. 1).

Aligning with the understanding that the complement system is involved in both initiation and contraction of immune responses 71, complement has also been shown to negatively regulate NLRP3 inflammasome activity: C1q suppresses caspase‐1 cleavage and subsequent mature IL‐1β production in human monocyte‐derived macrophages during the phagocytosis of apoptotic lymphocytes 72. The authors suggest that C1q may drive *NLRP12* mRNA expression and IL‐10 secretion, as both NLRP12 (via NF‐kB suppression) and IL‐10 (through activation of JAK signaling) are known to negatively regulate the NLRP3 inflammasome 73, 74 (Fig. 1). Thus by directly regulating NLRP3 inflammasome activation, C1q may limit the excessive NLRP3 inflammasome activation triggered by DAMPs (Fig. 1) released by late apoptotic cells 72. Given the merging role of C1q in inflammasome regulation, It may therefore be worthy to revisit the role of defective C1q activity observed in autoimmune disease such as in systemic lupus erythematosus 46 with an eye on this new C1q function.

### Cell metabolism as critical link between complement and NLRP3 inflammasome activation

Although complement, the TLRs, and the inflammasomes were initially discovered as pathogen sensors, it is now becoming increasingly clear that the ability of these systems to recognize an imbalance in normal cell metabolic processes and their capability to evoke appropriate reactive responses is of equal importance to cell homeostasis. For example, NLRP3 inflammasome priming and activation are strongly driven by increased glucose influx, heightened glycolysis, and increased ATP production, as demonstrated in human retinal tubular epithelial cells in diabetic nephrophaty 75, all events generally required for cell activation, proliferation, and effector function 8. Furthermore, both enzymes and products of glycolysis and the Krebs cycle have been shown to regulate NLRP3 inflammasome activity: the glycolytic enzyme pyruvate kinase M2 drives LPS‐induced NLRP3 activation in macrophages by regulating the hypoxia‐inducible factor 1 (HIF‐1α), a transcription factor that binds directly to the *IL1B* promoter and causes sustained production of this cytokine 76; and the Krebs cycle product succinate stabilizes HIF‐1α further, thereby supporting *IL1B* transcription 77. As a major glycolysis product, ATP is a strong driver of NLRP3 inflammasome assembly, as it activates P2X7 and induces IL‐1β and IL‐18 maturation and release 78. Also, adenosine itself sustains inflammasome activation induced by LPS, through stimulating the adenosine A(2A) receptor and cAMP/PKA/CREB/HIF‐1α pathway, a priming signal for IL‐1β production 79.

The NLRP3 inflammasome also integrates signals derived from AA and lipid metabolism in cells as well as ROS production, which is increased upon heightened mitochondrial activity required for cell effector functions. Activation of the AA sensor mammalian target of rapamycin complex 1 (mTORC1) has been demonstrated to be a potent NLRP3 inflammasome inducer in macrophages 80. Moreover, using the human monocytic cell line THP‐1, active NLRP3 inflammasomes have been shown to colocalize with mitochondria 11, where increased ROS generated by mitochondrial activity sustains NLRP3 inflammasome function 64.

Metabolic by‐products can also inhibit NLRP3 inflammasome activation. In particular, increased AMP generation activates the nutrient sensor AMP‐dependent protein kinase (AMPK) that impacts negatively on NLRP3 inflammasome function, as AMPK signaling promotes the switch from energy‐consuming processes such as glycolysis (which generally denotes states of high cellular activity) to oxidative metabolism associated with anti‐inflammatory and quiescent states and favors mitochondrial biogenesis and reduction in NLRP3 activation 41, 81, 82. Similarly, increased lactate, the main product of anaerobic glycolysis, is associated with reduced TLR4‐mediated inflammasome induction in monocytes and macrophages, through a pathway dependent on the lactate receptor Gi‐protein‐coupled receptor 81 and arrestin β‐2 83. The ketogenic metabolites β‐hydroxybutyrate 84, mono‐unsatured fatty acids (through AMPK signaling) 85, the N‐methyl‐d‐aspartate receptor for poly‐unsaturated fatty acids 86 and prostaglandin E2 87 have all been shown to inhibit inflammasome activity. Thus, the metabolic state of a cell modulates NLRP3 inflammasome function and metabolites produced by a cell during effector activity induce the inflammasome, while metabolites produced during quiescent, contracting, or tolerogenic cell responses inhibit inflammasome activity. Importantly, inflammasomes may not only integrate intracellular signals but also sense metabolic dysregulation on a systemic level, as the higher concentration of blood glucose found in T2D has been associated with NLRP3 inflammasome activation and high serum IL‐1β in patients 64.

**Figure 2:**
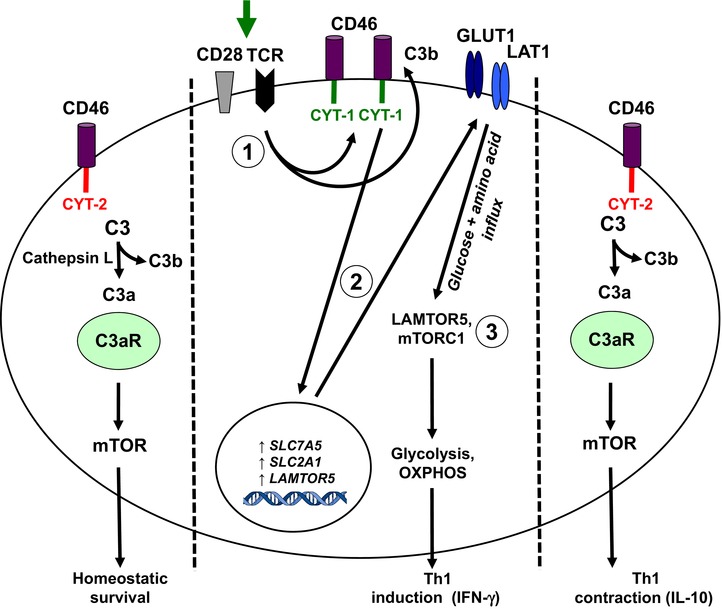
The role of C3 activation fragments in T‐cell homeostasis and in the induction of key metabolic events during Th1 responses. In resting T cells, the “tonic” generation of intracellular C3a via cathepsin L leads to the activation of the C3aR expressed on lysosomes and the low‐level activation of mTOR that sustains T‐cell survival (left). TCR activation and CD28 costimulation of resting T cells induces the local generation of the CD46 ligand C3b and increased expression of CD46 isoforms bearing CYT‐1 (1, middle). Autocrine CD46 CYT‐1‐driven signals then lead to upregulation of genes coding for the glucose transporter GLUT (*SLC2A1*), and the amino acid channel LAT1 (*SLC7A5*), allowing for increased influx of glucose and amino acids into the cell (2). In parallel, CD46 CYT‐1‐mediated signals induce increased expression of LAMTOR5, and via this assembly of the lysosome‐based machinery enabling amino acid sensing via mTORC1, which then leads the induction of glycolysis and OXPHOS required for IFN‐γ production (3). During Th1 contraction and induction of IL‐10 coexpression, CD46 isoform expression reverts to a CYT‐2 predominant pattern (through a mechanism that is currently unknown) and this is accompanied by reduced expression of GLUT1 and LAT1, downregulation of glycolysis and OXPHOS and reinstatement of C3a‐driven low level mTOR activity (right).

Although it is long known that systemic complement activation impacts on the function of metabolic organs (for an excellent review please see 88), autocrine complement activity can now also be connected with the regulation of “single cell” metabolism driving cell activation. For example, CD46 costimulation during T‐cell stimulation is required for metabolic reprogramming during Th1‐cell responses 13. CD46 is expressed in distinct isoforms (arising from differential splicing of a single gene) in CD4^+^ T cells and these isoforms differ in the expression of their cytoplasmic tails, termed CYT‐1 and CYT‐2. Both domains can transduce intracellular signals 52, 53, 71 and resting T cells mostly express CD46‐CYT‐2 13, 52, 53 (Fig. 2). Upon TCR stimulation, CYT‐1‐bearing CD46 isoforms are upregulated, engaged via T‐cell autocrine C3b production and induce the increased expression of the glucose and AA channels, GLUT1 and LAT1, respectively, thereby mediating the nutrient influx needed for T‐cell activation (Fig. 2). Moreover, CD46‐CYT‐1 also upregulates the late endosomal/lysosomal adaptor, MAPK and MTOR activator 5 (LAMTOR5), which then drives mTORC1 assembly and activation with subsequent increased glycolysis (Fig. 2) 13, in line with previous work that has linked augmented glycolysis in human CD4^+^ T cells with IFN‐γ production 89. CD46‐mediated signals also induce the switch from a high glycolytic state back to steady‐state glycolysis levels in CD4^+^ T cells and, via this, subsequently IL‐10 coproduction and finally Th1 contraction 13 (Fig. 2). This switch from high to low glycolysis is mediated by CD46 isoforms expressing CYT‐2, which become again the predominant CD46 isoforms in contracting T cells 13. The mechanism regulating CD46 isoform splicing or switching is not defined yet. Interestingly, CD46‐CYT‐1 also increases OXPHOS levels in activated T cells 13 and we are currently investigating the underlying CD46‐mediated signals driving this OXPHOS “burst” during T‐cell activation and its potential role in T‐cell homeostasis and/or memory development. The unexpected critical roles for complement and specifically CD46 in CD4^+^ T‐cell metabolic reprogramming is underpinned by the fact that T cells from CD46‐deficient patients have a defect in glycolysis, OXPHOS and Th1 (and Th17) induction 13. However, as a complement regulator serving as the murine homolog of CD46 in regards to Th1 induction and regulation has so far not been identified, these findings also show that there are substantial differences between species in the complement‐mediated signaling pathways regulating cell metabolism.

**Figure 3:**
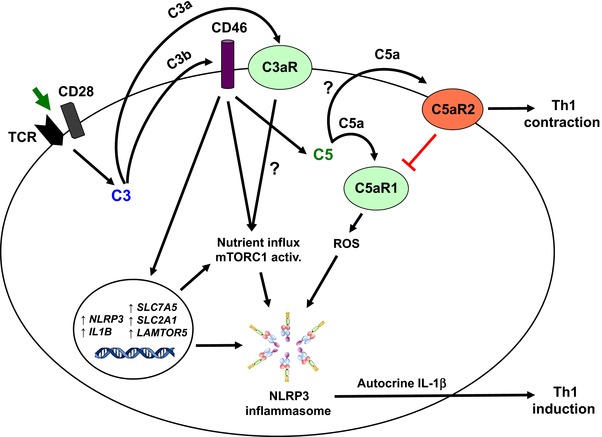
A complement‐metabolism‐inflammasome axis regulating human Th1 response induction and contraction. TCR stimulation of human CD4^+^ T cells induces the autocrine activation of CD46 and the C3aR via the “C3 system” that cumulates in nutrient influx, mTORC1 activation (which is a NLRP3 inflammasome activator) and induction of key metabolic events (see Fig. 2 for details). In addition, CD46 stimulation simultaneously induces gene expression of *NLRP3* and *IL1B* to prime the NLRP3 inflammasome as well as increased intracellular C5 activation and C5a generation (of note, the enzyme cleaving intracellular C5 into C5a and C5b has not yet been identified and is denoted by a question mark). Intracellularly generated C5a then engages the intracellular C5aR1 to amplify ROS production. Increased ROS levels, together with the indicated metabolic changes, induce the assembly of the NLRP3 inflammasome and subsequent IL‐1β (but not IL‐18) production required for optimal Th1‐cell induction. Cell surface expressed C5aR2 (engaged via secreted C5a/C5adesArg) negatively regulates C5aR1 signaling via a yet‐unidentified mechanism—and therefore controls the Th1‐cell responses.

Our recent work now suggests that autocrine complement activation‐driven metabolic changes are direct and critical upstream activators of the NLRP3 inflammasome in humans: We have now found that the canonical NLRP3 inflammasome surprisingly assembles not just in myeloid innate immune cells, but also in human activated CD4^+^ T cells, where it initiates caspase‐1‐dependent IL‐1β secretion and promotes IFN‐γ production and Th1 differentiation in an autocrine fashion 58 (Fig. 3). Importantly, NLRP3 assembly in these activated CD4^+^ T cells requires CD46 signaling, which not only induces *IL1B* gene transcription but also the increased intracellular generation of C5a and activation of the C5a receptor 1 (C5aR1). Intracellular C5aR1 engagement subsequently impacts on oxygen metabolism by inducing strong ROS generation and subsequent NLRP3 inflammasome activation (Fig. 3). Interestingly, CD46 and C5aR1‐driven NLRP3 activation in human CD4^+^ T cells induces IL‐1β but not IL‐18 production [58]. This is in contrast to mouse retinal epithelial and dendritic cells were complement activation is a strong inflammasome‐dependent inducer of both IL‐1β and IL‐18 61 and suggests that complement‐mediated production of these two cytokines is likely cell and/or context specific.

We also observed that CD46 and C5aR1‐driven NLRP3 activation is negatively controlled by surface‐expressed C5aR2 in an autocrine fashion, either through inhibition of intracellular C5aR1 activity or via a yet undefined mechanism (Fig. 3). We envisage that, whereas APC‐derived NLRP3‐activated IL‐1β supports initial Th1 priming, maintenance of the Th1 phenotype during differentiation and migration into the periphery may rely on the autocrine complement/NLRP3 cross‐talk. Importantly, we indeed observed that dysregulation of these autocrine pathways affects the inflammatory responses in autoimmune disease: CD4^+^ T cells from patients with CAPS, who express mutated, constitutively‐active NLRP3, and have increased autocrine IL‐1β secretion exhibit strongly increased Th1 responses that are normalized by treatment with the NLRP3 inflammasome‐specific inhibitor MCC950 58, 90.

Since mTORC1 activity has recently been identified as an NLRP3 inflammasome activator 80 and HIF‐1α stabilizer 91, we suggest a novel functional “complement‐metabolism‐inflammasome axis” in which autocrine complement‐mediated signals from C3 and C5 activation fragments drive *NLRP3* and *IL1B* transcription (Signal 1, via CD46 and/or C5aR1), but also provide Signal 2 via increased oxygen metabolism and ROS production (C5aR1) and additional induction of metabolic changes (mTORC1 assembly and glycolysis via CD46), all of which cumulate in NLRP3 activation, IL‐1β secretion and optimal Th1 induction (Fig. 3). Aligning with this model, we have observed that inhibition of mTOR by rapamycin abrogates NLRP3 activation and IL‐1β production in T cells, and reduces Th1‐cell induction (Arbore and Kemper, unpubl. obs.). Thus, the regulated cross‐talk between intracellularly activated complement components (the “complosome” [44, 58]), key metabolic pathways and the NLRP3 inflammasome emerges as fundamental to human Th1 induction and regulation. Although this new functional connection between these systems has so far only been shown in CD4^+^ T cells, we suggest that they also cooperate during cell activation and induction of effector function in other cells, including monocytes, APCs, neutrophils etc.

Furthermore, we found that T cells from CAPS patients have increased intracellular C5 expression and activation (Arbore et al., unpubl. obs.), which suggests that (intracellular) complement is not only an upstream inducer of NLRP3 inflammasome function but is, in turn, also regulated by NLRP3 activity. Similarly, the NLRP3 inflammsome not only senses metabolic changes but also contributes to the adaptions in glucose and triglyceride metabolism required during normal nutrient intake and energy expenditure (reviewed in 92). Thus, this novel complement‐metabolism‐inflammasome axis described here is likely part of a complex network in which all three systems engage in cross‐talk that is regulated by an intricate balance of positive and negative feedback loops. In this regard, it will be interesting to assess whether the complosome not only drives metabolism but is also able to sense metabolic changes and contribute to appropriate cellular adjustments.

## Conclusions

Accumulating evidence suggests that ancient pathogen‐sensing systems are also at the heart of “normal” cell activation and homeostasis and function via inducing and/or recognizing metabolic changes—and an emerging novel cross‐talk between (intracellular) complement as unexpected key regulator of cell metabolism and the NLRP3 inflammasome that senses changes in cellular metabolic states may be a critical contributor to this surveillance system. This notion suggests that the role of the complement system not only in autoimmune but also in metabolic diseases (including cancer) should be reevaluated—as a better understanding of the inducing and regulative mechanisms underlying these new roles for complement may deliver the foundation for novel therapeutic strategies combating these diseases.

## Conflict of interest

The authors declare no financial or commercial conflict of interest.

**Abbreviations**
AA: amino acid
AMPK: AMP‐dependent protein kinase
ASC: apoptosis speck protein
ATP: adenosine triphosphate
CAPS: cryopyrin‐associated periodic syndrome
CARD: caspase activation and recruitment domain
CR2: complement receptor 2
DAMP: danger‐associated molecular pattern
HSP90: heat shock protein 90
HIF‐1α: hypoxia‐inducible factor 1
MAC: membrane attack complex
MBL: mannose‐binding lectin
MCP: membrane cofactor protein
mTOR: mammalian target of rapamycin
NLR: Nod‐like receptor
OXPHOS: oxidative phosphorylation
PAMPs: pathogen‐associated molecular patterns
PRRs: pattern recognition receptors
PYHIN: Pyrin and HIN
RA: rheumatoid arthritis
RIG‐1: retinoic acid inducible gene 1
ROS: reactive oxygen species
SGT1: ubiquitin‐ligase suppressor of G2 allele of skp1
TLRs: toll‐like receptors
T2D: type‐2 diabetes
